# Synchronous adenocarcinomas of the colon presenting as synchronous colocolic intussusceptions in an adult

**DOI:** 10.1186/1477-7819-10-272

**Published:** 2012-12-15

**Authors:** Chuang-Wei Chen, Chieh-Wen Lai, Koung-Hong Hsiao

**Affiliations:** 1 Division of Colon and Rectal Surgery, Department of Surgery, Buddhist Tzu Chi General Hospital, No.289, Jianguo Rd., Sindian City, Taipei County, 231, Taiwan (R.O.C; 2 School of Medicine, Tzu Chi University, Hualien, Taiwan; 3 Division of General Surgery, Department of Surgery, Buddhist Tzu Chi General Hospital, Taipei Branch, Taipei, Taiwan

**Keywords:** Intussusception, Colon, Synchronous

## Abstract

Intussusception is uncommon in adults. To our knowledge, synchronous colocolic intussusceptions have never been reported in the literature. Here we described the case of a 59-year-old female of synchronous colocolic intussusceptions presenting as acute abdomen that was diagnosed by CT preoperatively. Laparotomy with radical right hemicolectomy and sigmoidectomy was undertaken without reduction of the invagination due to a significant risk of associated malignancy. The final diagnosis was synchronous adenocarcinoma of proximal transverse colon and sigmoid colon without lymph nodes or distant metastasis. The patient had an uneventful recovery. The case also emphasizes the importance of thorough exploration during surgery for bowel invagination since synchronous events may occur.

## Background

Intussusception accounts for only 1 to 5% of intestinal obstructions in adults [1]. Intussusception occurs most commonly in the small bowel, and colonic intussusception accounts for 15 to 27% of its occurrence [2-4]. Synchronous intussusception occurs even more rarely, and is only sporadically reported in the literature.

## Case presentation

**Figure 1:**
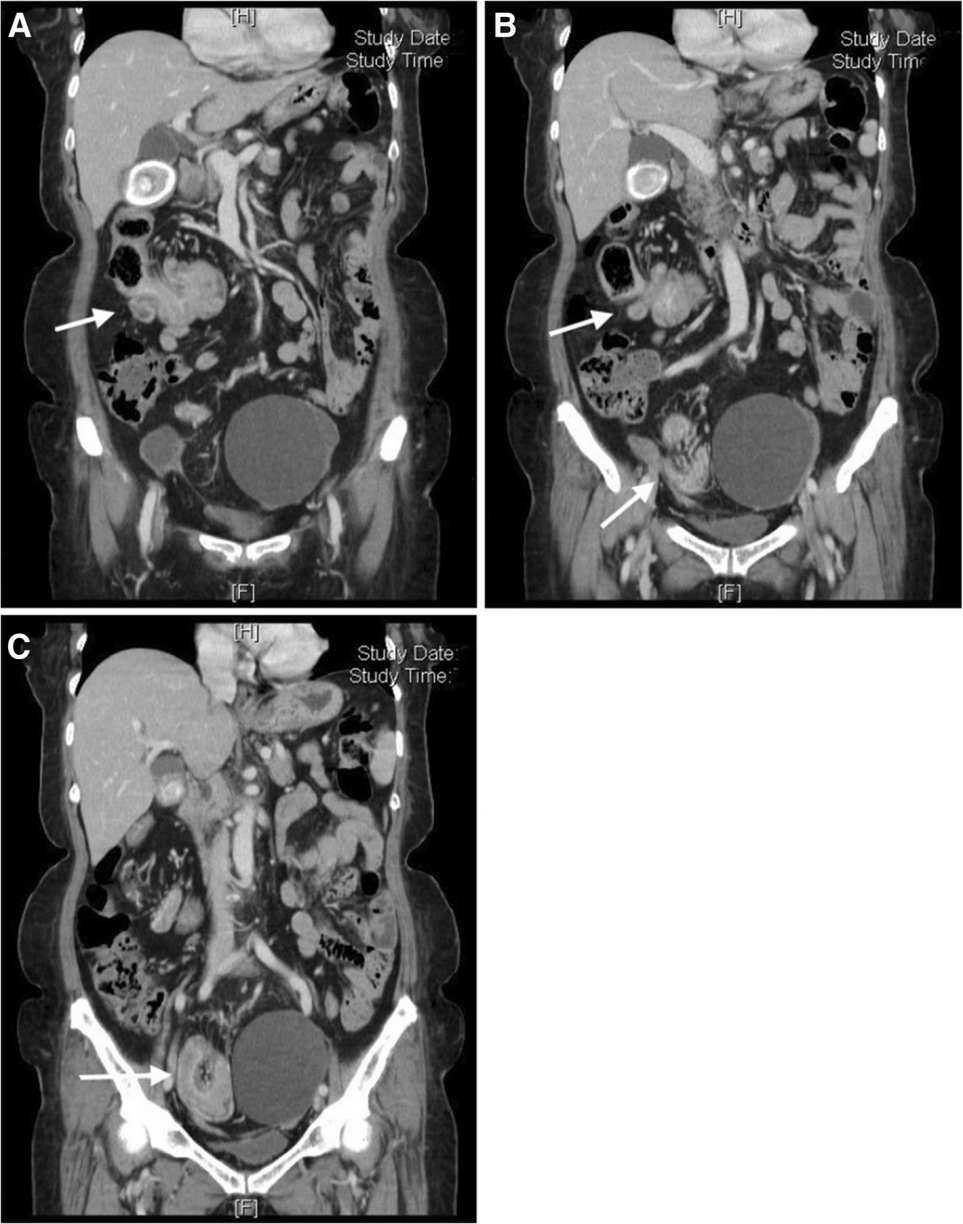
Computer tomography (CT) (arrows) showed the characteristic signs of the edematous bowel wall and mesentery within the lumen at the proximal transverse colon (A and B) and the rectosigmoid junction (B and C).

**Figure 2:**
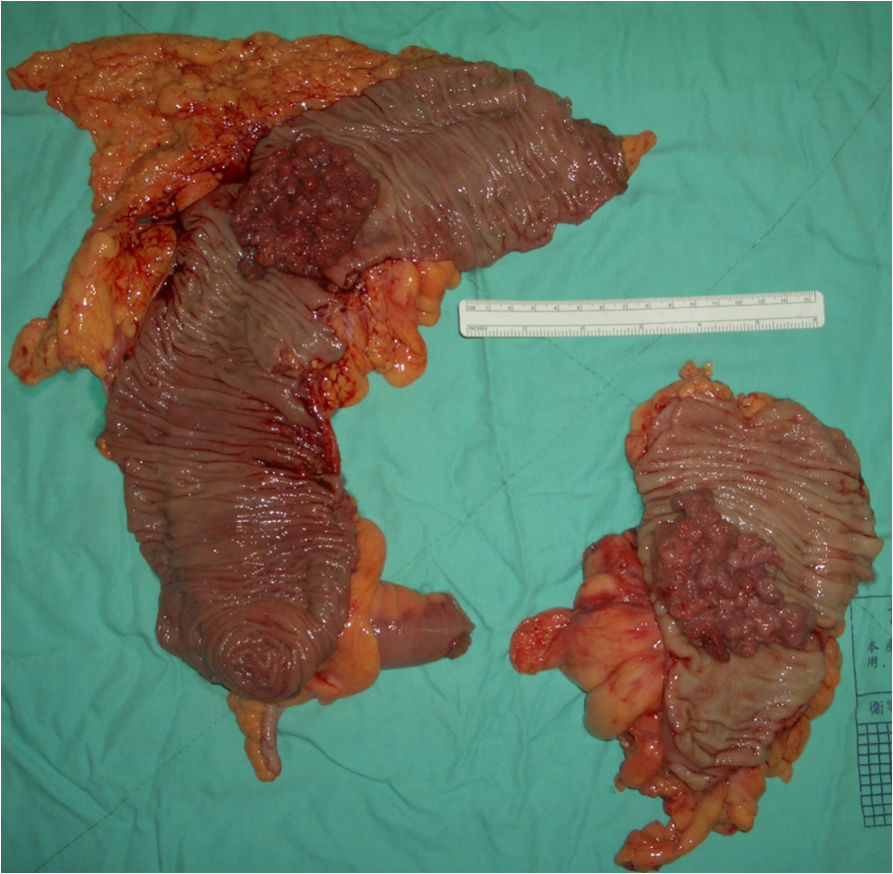
The surgical specimens showed synchronous tumors at the proximal transverse colon and sigmoid colon.

A 59-year-old woman presented with an eight-hour history of progressive abdominal pain and vomiting. She had occasionally experienced abdominal pain, diarrhea, and rectal bleeding over the past 2 years. Her past medical history included hysterectomy due to uterine myoma for 10 years. At the emergency department, she was alert and appeared to be in moderate distress. Her vital signs were stable and her temperature was 37.2°C. On physical examination, her abdomen was soft but moderately tender to palpation. Digital rectal examination did not reveal any mass or blood. Laboratory test results showed anemia (hemoglobin 9.0 g/dl). Abdominal radiography was unremarkable. Due to her progressively worsening abdominal pain, computer tomography (CT) of the abdomen was performed and the result revealed synchronous colocolic intussusceptions at the rectosigmoid junction and proximal transverse colon, as well as ovarian cystic lesions (Figure 1A-C). The intussusception was indicated by the characteristic sign of edematous bowel wall and mesentery within the lumen. Synchronous intussusceptions were identified by laparotomy. Radical right hemicolectomy and sigmoidectomy were undertaken because of the high risk of malignant etiology in the colonic intussusception. Left oophorocystectomy was also performed (Figure 2). No reduction of the invagination was attempted due to the high risk of malignant pathology. The final diagnosis was synchronous moderately differentiated adenocarcinoma of the proximal transverse colon and sigmoid colon, tumor, node, metastasis (TNM) stage, T3, N0, without distant metastasis. Twenty lymph nodes were retrieved in the specimen from the right colectomy and twelve nodes in the specimen from sigmoidectomy. The pathology analysis showed the specimen from the oophorocystectomy to be a benign simple cyst. The patient had an uneventful recovery. She received adjuvant chemotherapy with oral UFUR (Tegafur 100 mg, uracil 224 mg) due to high risk features of systemic recurrence of pathological disease, including positive lymphovascular invasion and perineural invasion (according to the NCCN clinical practice guidelines for oncology, V.1, 2010). No recurrent symptoms or signs were noted at follow-up after 12 months.

## Discussion

Adult intussusception represents only 5% of all cases of intussusception [5,6]. Unlike children, more than 90% of all adult intussusceptions have pathological causes, especially colonic intussusception [7]. Malignant intussusceptions account for the majority of colonic invaginations, whereas 80% of neoplasms associated with small bowel intussusception were benign [2]. Primary colon adenocarcinoma is the most common underlying malignant lesion in colonic intussusception [2]. Due to the high malignancy rate, en bloc resection without reduction for adult colonic intussusception is advocated to prevent any potential spread of the tumor and contamination of the abdominal cavity due to incidental perforation [8,9]. Regarding benign tumors, lipoma is the most commonly pathologic lead point in both small and large bowel intussusception. The recognition of the tumor spectrum and incidence of malignances differ between small and large bowel intussusception in adults can help surgeons adopt appropriate surgical procedure for intussusception.

In contrast to pediatric presentation of acute intussusception, the classic triad of cramping abdominal pain, bloody diarrhea and a palpable tender mass is rare in adults [3]. The symptoms of adult intussusception are non-specific [3]. Abdominal pain is the most common presenting complaint, followed by nausea, vomiting, gastrointestinal bleeding and diarrhea [2]. Morera Ocon, *et al*. [4] reported that the preoperative diagnosis of invagination was made in 25/30 (83%) of patients. However, in a review by Athanasios Marinis, *et al*. [3], the authors stated that variability in clinical presentation and imaging features often make the preoperative diagnosis of intussusception a challenging and difficult task. Reijene, *et al*. [10] reported a preoperative diagnostic rate of 50%, while Eisen, *et al*. [11] reported a lower rate of 40.7%.

Abdominal radiographs usually demonstrate signs of intestinal obstruction and may provide information about the site of the obstruction [3]. Upper gastrointestinal contrast series or barium enema examination may demonstrate the characteristic appearance of a coiled-spring or cup-shaped filling defect [3]. Ultrasonography is considered a useful tool for the diagnosis of intussusception by an experienced observer. The typical imaging features include the target or doughnut signs on the transverse view and the pseudo-kidney sign on the longitudinal view [3]. With the growing use of CT in the diagnosis of abdominal diseases, especially for the patients presenting with acute abdomen, preoperative detection of intussusception has increased. Most studies also indicate that CT is the most accurate diagnostic tool for intussusception [12]. Typical features of intussusception on CT s include the pseudo-kidney, target or bulls-eye sign, or the appearance of bowel-within-bowel configuration with or without fat and mesenteric vessels. The CT scan may also define the location, the nature of the mass, its relationship to surrounding tissues and additionally, it may help in disease staging in patients in whom malignancy is the suspected cause of the intussusception [13,14].

## Conclusions

To our knowledge, synchronous colocolic intussusceptions have never been reported in the literature. Here we describe the first case of synchronous colocolic intussusceptions caused by synchronous adenocarcinoma of the colon diagnosed preoperatively by CT. The case also emphasizes the importance of thorough exploration during surgery for bowel invagination, since synchronous events may occur.

## Consent

Written informed consent was obtained from the patient for publication of this report and any accompanying images.

## Competing interests

The authors declare that they have no competing interests.

## Authors’ contribution

We declare that all the listed authors have participated actively in the study and all meet the requirements of the authorship. CWC designed the study and wrote the protocol, KHH performed research/study, CWL managed the literature searches and analyses, CWC wrote the first draft of the manuscript. All authors read and approved the final manuscript.
